# Prevalence of Thumb and Wrist Pain Among Smartphone Users in the Saudi Arabian General Population: A Cross-Sectional Study

**DOI:** 10.7759/cureus.52922

**Published:** 2024-01-25

**Authors:** Mahmoud Mohammed Hassaan, Mohammad A Jareebi, Hanan A AlKaabi, Alhassan H Hobani, Yazeed M Alfuhigi, Norah K Albahli, Hatim Alrashed, Sami K Alotaibi, Abdulaziz S Almadi, Othman A Iskander, Khalid Alyahyawi, Jamaludeen A Othman, Waseem S Borik, Mohammed Y Qaarie

**Affiliations:** 1 Division of Orthopedic, Department of Surgery, Jazan University, Jazan, SAU; 2 Department of Community and Family Medicine, Jazan University, Jazan, SAU; 3 College of Medicine, King Khalid University, Abha, SAU; 4 College of Medicine, Jazan University, Jazan, SAU; 5 College of Medicine, Jouf University, Sakaka, SAU; 6 College of Medicine, Princess Nourah bint Abdulrahman University, Riyadh, SAU; 7 Department of Medicine and Surgery, College of Medicine, Jouf University, Sakaka, SAU; 8 Department of Orthopedics, College of Medicine, Jouf University, Sakaka, SAU; 9 Department of Internal Medicine, Qassim University, Qassim-Buraydah, SAU; 10 Department of Surgery, Jazan University, Jazan, SAU; 11 Department of Surgery, Faculty of Medicine, Jazan University, Jazan, SAU; 12 Department of Anesthesia and Critical Care, Jazan University Hospital, Jazan, SAU; 13 Department of Anesthesia and Critical Care, Jazan University, Jazan, SAU

**Keywords:** middle east, saudi arabia, thumb pain, upper limb disorders, musculoskeletal disorders, smartphone addiction, problematic smartphone use

## Abstract

Background

This study aims to investigate the prevalence of thumb and wrist pain among smartphone users in the general population of Saudi Arabia, examining the potential links between the duration and frequency of smartphone usage, specific smartphone activities, and the occurrence of discomfort in the thumb and wrist.

Methodology

This descriptive cross-sectional study was conducted at Jazan University, Saudi Arabia, between July 2022 and June 2023. The questionnaire developed following an extensive literature review, covered demographic information, smartphone usage patterns, assessment of thumb or wrist pain, and evaluation of the impact of this discomfort. Descriptive statistical methods were employed, and relationships between thumb or wrist pain and demographic variables were analyzed using chi-square and Wilcoxon rank-sum tests.

Results

In total, 811 participants were included in this study. Approximately 322 (39.7%) study participants reported wrist or thumb pain. Notably, female sex (*P* = 0.001) and prolonged daily smartphone usage exceeding five hours (*P* = 0.045) were linked to thumb or wrist pain. Additionally, emailing on smartphones was linked to thumb or wrist pain (*P *= 0.04). Concerning smartphone ergonomics, the majority of respondents reported holding their phones with one hand (215, 66.77%), in a downward position (290, 90.06%), and with their wrists bent downward (136, 42.24%).

Conclusions

Our study highlights a relatively high prevalence of wrist and thumb pain among smartphone users in the general Saudi Arabian population. Furthermore, we identified a connection between prolonged smartphone use and wrist and thumb pain, with a more pronounced prevalence observed among females.

## Introduction

The ubiquity of smartphone usage has surged significantly in recent years, reaching 6.8 billion users globally in 2020, constituting 68% of the world population [1]. Notably, in Saudi Arabia, this prevalence is even more pronounced, with an astounding 97% of adolescents owning smartphones [2].

Despite the manifold advantages smartphones offer, including productivity enhancement, streamlined information retrieval, and enhanced social interactions [3], they have been intricately linked to overuse behavior, termed problematic smartphone use [4]. This behavior, characterized by smartphones interfering with daily life, can escalate to smartphone addiction - a nonchemical behavioral addiction involving human-machine interaction [5]. Globally, smartphone addiction affects 26.99% of the population, with particularly high rates in the Eastern Mediterranean region, including Saudi Arabia, owing to the country's affluence, technological advancement, and high Human Development Index [6,7].

Excessive smartphone usage not only poses physiological challenges, such as headaches, eye strain, and musculoskeletal problems but also contributes to psychological complications, including sleep disturbances, anxiety, and depression [8]. These complications, in turn, can detrimentally impact concentration, academic performance, and interpersonal skills [9].

The frequent use of electronic devices requiring thumb involvement can exacerbate thumb strain [10,11]. Additionally, awkward postures during device usage elevate the risk of musculoskeletal disorders [12]. The pain experienced in the thumb and wrist due to frequent smartphone app interactions can intensify thumb and wrist discomfort, as screen interaction necessitates intricate thumb and finger movements [13]. Numerous studies have provided empirical evidence that this overuse can result in median nerve enlargement, thumb pain, reduced pinch strength, and diminished hand functionality [14,15], ultimately heightening the risk of carpal tunnel syndrome [16], thereby collectively contributing to a diminished quality of life [17].

While various studies in Saudi Arabia have explored different facets of smartphone overuse, the majority have centered around university students, with none specifically delving into the prevalence of wrist or thumb pain in the general population. Hence, the primary objective of this study is to comprehensively assess the prevalence and severity of thumb and wrist pain among smartphone users in the general populace. Furthermore, the study seeks to meticulously explore the intricate correlations between smartphone usage duration and frequency, specific smartphone activities, and the development of thumb and wrist pain.

## Materials and methods

This cross-sectional study was carried out at the University of Jazan within the general population of Saudi Arabia from July 2022 to June 2023, excluding individuals with cognitive impairments, those unable to complete the questionnaire, and those who declined to participate. The sample size determination utilized the Epi Info program [18], resulting in a required minimum sample size of 385. This calculation was based on a 5% margin of error, a 95% confidence level, and a population of 34,110,821 in 2022, as per records from the General Authority for Statistics.

Data collection methods

The questionnaire, developed following an extensive literature review, was initially formulated in English and subsequently translated into Arabic. A panel of experts reviewed and approved the questionnaire. Subsequently, a pilot study involving 20 participants was conducted to enhance the questionnaire's clarity and comprehensibility based on participant feedback. Following this refinement, data collection commenced. Employing a non-probability convenient sampling approach, an online self-administered questionnaire was disseminated via social media.

The questionnaire consists of four sections: demographic data, smartphone usage, assessment of thumb or wrist pain, and evaluation of the impact of thumb or wrist pain. The demographic data section includes age, gender, nationality, educational attainment, occupation, monthly income, and residential area. The smartphone usage section encompasses variables such as the annual and daily duration of smartphone use, frequency of smartphone utilization, and common activities undertaken while using smartphones. The thumb/wrist pain assessment section collects data on the history of thumb or wrist pain within the past six months, the frequency and severity of such pain, seeking medical treatment, and ergonomic factors related to smartphone usage, referring to positions adopted during smartphone use. The section concerning the impact of thumb or wrist pain assesses its effect on daily activities and overall quality of life.

Our study received approval from the Scientific Research Ethics Committee (REC) at Jazan University in Saudi Arabia (reference number REC-45/02/734) by the 1964 Helsinki Declaration and its subsequent amendments or comparable ethical standards. Informed consent was obtained from all participants, and their information was maintained confidentially, with the data solely utilized for scientific purposes.

Data analysis

The collected data underwent cleaning and organization in an Excel spreadsheet before being imported into R software version 4.2.2. Descriptive statistics were used to analyze continuous data, employing mean and standard deviation, while categorical data were summarized with frequencies and percentages. Chi-square and Wilcoxon rank-sum tests were used to assess the relationship between thumb or wrist pain and demographic characteristics. The chi-square test was used to investigate the association between thumb or wrist pain and variables such as duration, frequency of smartphone usage, and smartphone activities. Furthermore, the chi-square test was utilized to examine the association between the severity of wrist or thumb pain and respondents' quality of life and its relationship with duration, frequency of smartphone use, and smartphone activities.

## Results

In the study, a total of 811 participants, with a median age of 26 years, were included. The majority of participants were female (536, 66.09%), held a university degree (623, 76.82%), earned 5,000 Saudi riyals or less (444, 54.75%), and resided in the central region of Saudi Arabia (338, 41.68%). Detailed demographic characteristics are presented in Table 1.

**Table 1:** Sociodemographic characteristics of the study participants (n = 811). SR, Saudi riyal

Characteristics	Overall (*n* = 811), *n* (%)	Thumb or wrist pain		*P*-value
		No (*n *= 489)	Yes (*n *= 322)	
Gender				
Female	536 (66.09)	302 (61.76)	234 (72.67)	0.001
Male	275 (33.91)	187 (38.24)	88 (27.33)	
Age, median (IQR)	26 (22-39.50)	25 (22-40)	26 (22-39)	0.914
Marital status				
Single	488 (60.17)	294 (60.12)	194 (60.25)	0.087
Married	295 (36.37)	184 (37.63)	111 (34.47)	
Divorced	26 (3.21)	10 (2.04)	16 (4.97)	
Widowed	2 (0.25)	1 (0.20)	1 (0.31)	
Educational level				
Primary school	8 (0.99)	4 (0.82)	4 (1.24)	0.092
Intermediate school	18 (2.22)	10 (2.04)	8 (2.48)	
Secondary school	162 (19.98)	98 (20.04)	64 (19.88)	
University degree	623 (76.82)	377 (77.10)	246 (76.40)	
Nationality				
Non-Saudi	83 (10.23)	51 (10.43)	32 (9.94)	0.885
Saudi	728 (89.77)	438 (89.57)	290 (90.06)	
Income Level				
5,000 or less SR	444 (54.75)	275 (56.24)	169 (52.48)	0.613
5,000-9,999 SR	153 (18.87)	89 (18.20)	64 (19.88)	
10,000-14,999 SR	112 (13.81)	62 (12.68)	50 (15.53)	
More than 15,000 SR	102 (12.58)	63 (12.88)	39 (12.11)	
Occupation				
Healthcare worker	82 (10.11)	48 (9.82)	34 (10.56)	0.466
Non-healthcare worker	217 (26.76)	133 (27.20)	84 (26.09)	
Students in medical field	147 (18.13)	96 (19.63)	51 (15.84)	
Student in nonmedical field	88 (10.85)	46 (9.41)	42 (13.04)	
Not working	277 (34.16)	166 (33.95)	111 (34.47)	
Residence area				
Central	338 (41.68)	197 (40.29)	141 (43.79)	0.471
Northern	116 (14.30)	78 (15.95)	38 (11.80)	
South	66 (8.14)	43 (8.79)	23 (7.14)	
Eastern	131 (16.15)	74 (15.13)	57 (17.70)	
West	160 (19.73)	97 (19.84)	63 (19.57)	

Concerning the prevalence of thumb or wrist pain, 322 (39.7%) participants reported experiencing it. A significant association was observed between female gender and thumb or wrist pain (*P*-value = 0.001), and using smartphones for more than five hours a day was also associated with this pain (*P*-value = 0.045). Specifically, engaging in smartphone emailing activities was linked to thumb or wrist pain (*P*-value = 0.04) (Table 2).

**Table 2:** Association between wrist or thumb pain with duration, frequency of smartphone use, and smartphone activities (n = 811).

Characteristics	Thumb or wrist pain		*P*-value
	No (*n* = 489)	Yes (*n* = 322)	
How long have you been using a smartphone?			
3 years and less	16 (3.27%)	8 (2.48%)	0.812
4-9 years	140 (28.63%)	87 (27.02%)	
10-15 years	174 (35.58%)	113 (35.09%)	
More than 15 years	159 (32.52%)	114 (35.40%)	
How many hours do you use your smartphone?			
1-2 hours	21 (4.29%)	8 (2.48%)	0.045
2-3 hours	64 (13.09%)	28 (8.70%)	
3-5 hours	147 (30.06%)	89 (27.64%)	
> 5 hours	257 (52.56%)	197 (61.18%)	
Frequency of smartphone use in a day			
Every hour	300 (61.35%)	184 (57.14%)	0.241
Every minute	189 (38.65%)	138 (42.86%)	
Which activities do you commonly engage in when using your smartphone?			
Texting	356 (72.80%)	238 (73.91%)	0.722
Emailing	103 (21.06%)	88 (27.33%)	0.040
Social media	432 (88.34%)	287 (89.13%)	0.747
Web	218 (44.58%)	163 (50.62%)	0.092
Gaming	162 (33.13%)	111 (34.47%)	0.755
Watching videos	271 (55.42%)	187 (58.07%)	0.569

Regarding the ergonomics of smartphone use, the majority of participants reported holding their phones with one hand (215, 66.77%), in a downward position (290, 90.06%), and with their wrists bent downward (136, 42.24%) (Table 3).

**Table 3:** Ergonomic factors of thumb or wrist pain among smartphone users (n = 322).

Characteristics	*n* (%)
How often does the thumb or wrist pain?	
Frequently	48 (14.91)
Occasionally	155 (48.14)
Rarely	119 (36.96)
Do you sought any medical treatment?	
No	278 (86.34)
Yes	44 (13.66)
Do you hold your smartphone with one hand?	
Both hands	107 (33.23)
One hand	215 (66.77)
Do you hold your smartphone in a lying down position?	
No	32 (9.94)
Yes	290 (90.06)
Describe the posture of your wrist	
Bent downward	136 (42.24)
Bent upward	115 (35.71)
Straight	71 (22.05)
Have you made any adjustments to alleviate thumb or wrist pain?	
No	59 (18.32)
Yes	263 (81.68)
To what extent does the thumb or wrist pain?	
Not at all	103 (31.99)
Mild	135 (41.93)
Moderate	70 (21.74)
Severe	14 (4.35)
Does the thumb or wrist pain affect your quality of life?	
Not at all	133 (41.30)
Slightly	129 (40.06)
Moderately	48 (14.91)
Significantly	12 (3.73)

The duration of smartphone use was not found to be associated with the severity of thumb or wrist pain (*P* > 0.05). However, playing smartphone games was significantly linked to the severity of thumb or wrist pain (*P* = 0.031) (Table 4).

**Table 4:** Association of wrist or thumb pain severity and duration, frequency of smartphone use, and smartphone activities (n = 322).

Characteristics	Thumb or wrist pain severity					*P*-value
	Extremely severe (*n* = 29)	Severe pain (*n* = 57)	Moderate pain (*n* = 118)	Mild pain (*n* = 97)	No pain (*n* = 21)	
How long have you been using a smartphone?						
3 years and less	1 (3.45%)	1 (1.75%)	3 (2.54%)	2 (2.06%)	1 (4.76%)	0.445
4-9 years	7 (24.14%)	13 (22.81%)	28 (23.73%)	31 (31.96%)	8 (38.10%)	
10-15 years	10 (34.48%)	17 (29.82%)	53 (44.92%)	29 (29.90%)	4 (19.05%)	
More than 15 years	11 (37.93%)	26 (45.61%)	34 (28.81%)	35 (36.08%)	8 (38.10%)	
How many hours do you use your smartphone?						
1-2 hours	0 (0.00%)	1 (1.75%)	2 (1.69%)	4 (4.12%)	1 (4.76%)	0.335
2-3 hours	3 (10.34%)	4 (7.02%)	15 (12.71%)	5 (5.15%)	1 (4.76%)	
3-5 hours	7 (24.14%)	16 (28.07%)	26 (22.03%)	36 (37.11%)	4 (19.05%)	
>5 hours	19 (65.52%)	36 (63.16%)	75 (63.56%)	52 (53.61%)	15 (71.43%)	
What activities do you commonly engage in when using your smartphone?						
Texting	17 (58.62%)	41 (71.93%)	93 (78.81%)	72 (74.23%)	15 (71.43%)	0.354
Email	5 (17.24%)	20 (35.09%)	39 (33.05%)	20 (20.62%)	4 (19.05%)	0.080
Social media	22 (75.86%)	52 (91.23%)	107 (90.68%)	88 (90.72%)	18 (85.7%)	0.224
Web browsing	11 (37.93%)	32 (56.14%)	59 (50.00%)	52 (53.61%)	9 (42.86%)	0.535
Gaming	3 (10.34%)	23 (40.35%)	47 (39.83%)	33 (34.02%)	5 (23.81%)	0.031
Watching videos	11 (37.93%)	31 (54.39%)	73 (61.86%)	60 (61.86%)	12 (57.1%)	0.224

Furthermore, moderate daily activity interference and a slight impact on the quality of life were associated with more extreme severity of thumb or wrist pain (*P* < 0.001). Not seeking medical treatment for the pain was associated with greater extreme severity (*P* = 0.013), and adjusting to alleviate the pain was also linked to more extreme severity (*P* = 0.009) (Table 5).

**Table 5:** Association of wrist or thumb pain severity and respondents’ quality of life (n = 322).

Characteristics	Thumb or wrist pain severity					*P*-value
	Extremely severe (*n* = 29)	Severe pain (*n* = 57)	Moderate pain (*n* = 118)	Mild pain (*n* = 97)	No pain (*n* = 21)	
To what extent does the thumb or wrist pain interfere with your daily activity?						
Not at all	3 (10.34%)	2 (3.51%)	34 (28.81%)	52 (53.6%)	12 (57.1%)	<0.001
Mild interference	8 (27.59%)	25 (43.86%)	56 (47.46%)	40 (41.4%)	6 (28.5%)	
Moderate interference	12 (41.38%)	25 (43.86%)	25 (21.19%)	5 (5.15%)	3 (14.2%)	
Severe interference	6 (20.69%)	5 (8.77%)	3 (2.54%)	0 (0.00%)	0.00	
Thumb or wrist pain affects the quality of your life						
Not at all	4 (13.79%)	5 (8.77%)	48 (40.68%)	62 (63.9%)	14 (66.7%)	<0.001
Slightly	11 (37.93%)	30 (52.63%)	54 (45.76%)	30 (30.9%)	4 (19.05%)	
Moderately	9 (31.03%)	16 (28.07%)	15 (12.71%)	5 (5.15%)	3 (14.29%)	
Significantly	5 (17.24%)	6 (10.53%)	1 (0.85%)	00	00	
Do you seek any medical treatment?						
No	19 (65.52%)	50(87.72%)	103 (87.29%)	86 (88.6%)	20 (95.2%)	0.013
Yes	10 (34.48%)	7 (12.28%)	15 (12.71%)	11 (11.3%)	1 (4.76%)	
Have you made any adjustments to alleviate the thumb or wrist pain?						
No	3 (10.34%)	9 (15.79%)	21 (17.80%)	16 (16.5%)	10 (47.6%)	0.009
Yes	26 (89.66%)	48 (84.21%)	97 (82.20%)	81 (83.5%)	11 (52.38%)	

## Discussion

Over the past decade, smartphone usage has witnessed a substantial surge due to its multifunctionality and convenience. A study conducted in Riyadh, Saudi Arabia, reported that 76% of participants exhibited a moderate to high risk of smartphone and internet addiction [19]. This excessive smartphone use has adverse physical, cognitive, and psychological effects, including musculoskeletal disorders [10,20,21]. This study investigates the relationship between smartphone use and thumb/wrist pain in the Saudi Arabian population.

Our study primarily included single female participants with university-level education, aligning with demographics observed in previous Saudi Arabian and Malaysian studies [11,22-24]. However, our research differs by encompassing a broader demographic, with participants having a mean age in their late twenties. Most prior studies focused on university students, typically in their early twenties [11,25,26]. Another similar study in Saudi Arabia examined the general population but was restricted to Riyadh residents [27].

Approximately 322 (39.7%) participants reported wrist/thumb pain. In comparison to localized studies in Saudi Arabia, this prevalence is notably higher than that reported among students in Majmaah (13%) and Jeddah (20%) but lower than that found in the general Riyadh population (60%) [11,22,27]. Our study's prevalence falls within the range reported in Bangladesh and India (24%-38%) but is higher than in China (8%) and lower than in Malaysia (70%-80%) [24,28,29]. Contrasted with a systematic review by Zirek et al. [30] (13%-32%), our findings reveal a high prevalence of wrist/thumb pain in Saudi Arabia. Furthermore, we found a link between prolonged smartphone use and wrist/thumb pain, consistent with results from a study among the general population in Riyadh city [27] and among students in Saudi Arabia (Jeddah and Makkah) and Brazil [22,25,31].

Excessive smartphone use can lead to wrist/thumb pain through various mechanisms. Sustained smartphone holding with awkward wrist positions, such as flexion and ulnar deviation, can subject the wrist joint to excessive strain. This can subsequently increase pressure within the carpal tunnel, resulting in compression of its contents, particularly affecting the median nerve and the flexor pollicis longus tendon, leading to musculoskeletal symptoms in the hand [32]. Carpal tunnel syndrome is specifically linked to excessive smartphone use [33]. Repetitive movements with a static wrist posture, like scrolling and texting using the fingers, can increase the risk of microscopic muscular damage, resulting in hand pain, especially in the thumb (the most frequently used finger with a smartphone). Such muscular damage can impair fine motor functions in the fingers [34,35]. Additionally, muscle overuse is associated with a reduced pain threshold and muscular fatigue [36,37].

Our study found a significantly higher prevalence of wrist/thumb pain among females, consistent with studies conducted in Jeddah city and Singapore [22,26]. This gender-related association may be attributed to biological and psychological factors. Females have distinct musculoskeletal architecture, metabolic functions, and hormonal influences compared to males. Additionally, females are more sensitive and likely to report their symptoms [22,26]. However, a study among university students in Brazil did not find a statistically significant difference in pain scores between males and females [31].

Our study stands out due to several unique features. It is the first study to investigate the association between wrist/thumb pain and smartphone use among the entire Saudi Arabian general population. We conducted the study on a large scale, enrolling a substantial number of participants, ensuring better representativeness, and enhancing the generalizability of our findings. We employed a simple and user-friendly questionnaire format, encouraging greater participation. However, the convenience sampling method used for data collection may limit our study's scope. Our study is also strengthened by providing a detailed wrist/thumb pain assessment, including pain severity, frequency, and treatment-seeking behavior. Many previous studies lacked this level of pain analysis.

Nevertheless, recall bias is possible, which may affect the confidence in our findings. Therefore, we recommend conducting longitudinal studies to explore this association further. Additionally, we suggest conducting similar large-scale research to investigate other musculoskeletal issues (e.g., shoulder, neck, and back) and the use of other digital devices (e.g., laptop video games). Based on our study's findings, national educational programs promoting safe smartphone use and the prevention of musculoskeletal disorders could be developed.

## Conclusions

Our study reveals a relatively high incidence of wrist/thumb pain among smartphone users in the general Saudi Arabian population. We have identified an association between prolonged smartphone use and wrist/thumb pain, with a greater prevalence observed among females. Our findings provide a scientific foundation for the development of national educational initiatives aimed at promoting safe smartphone usage and preventing musculoskeletal disorders. We recommend further large-scale research to explore other musculoskeletal conditions and the use of various digital devices.
